# Correlation of Serum Cystatin C with Glomerular Filtration Rate in Patients Receiving Platinum-Based Chemotherapy

**DOI:** 10.1155/2016/4918325

**Published:** 2016-12-18

**Authors:** Ernesta Cavalcanti, Vittoria Barchiesi, Dionigio Cerasuolo, Flaviano Di Paola, Monica Cantile, Sabrina Chiara Cecere, Sandro Pignata, Alessandro Morabito, Raffaele Costanzo, Massimo Di Maio, Francesco Perrone

**Affiliations:** ^1^Laboratory Medicine Unit, Department of Diagnostic Pathology and Laboratory, Istituto Nazionale Tumori “Fondazione G. Pascale”, IRCCS, Naples, Italy; ^2^Uroginecologic Department, Istituto Nazionale Tumori “Fondazione G. Pascale”, IRCCS, Naples, Italy; ^3^Thoraco-Pulmonary Medical Oncology Unit, Istituto Nazionale Tumori “Fondazione G. Pascale”, IRCCS, Naples, Italy; ^4^Clinical Trials Unit, Istituto Nazionale Tumori “Fondazione G. Pascale”, IRCCS, Naples, Italy; ^5^Department of Oncology, University of Turin, AOU San Luigi Gonzaga, Orbassano, Italy

## Abstract

*Objectives*. Serum cystatin C seems to be an accurate marker of glomerular filtration rate (GFR) compared to serum creatinine. The aim of this work was to explore the possibility of using serum cystatin C instead of serum creatinine to early predict renal failure in cancer patients who received platinum based chemotherapy.* Design and Methods*. Serum creatinine, serum cystatin C concentrations, and GFR were determined simultaneously in 52 cancer patients received carboplatin-based or cisplatin-based chemotherapy. Serum creatinine was assayed on Cobas C6000-Roche, serum cystatin C assay was performed on AIA 360-Tosoh, and GFR was determined in all patients, before the first cycle of chemotherapy and before the subsequent administrations.* Results*. In the overall series, for the prediction of a fall of GFR < 80 mL/min/1.73 m^2^, the AUC of the ROC curve for cystatin C was 0,667 and the best threshold was 1.135 mg/L (sensitivity 90.5%, specificity 61.1%). For a GFR fall < 60 mL/min/1.73 m^2^, the AUC of ROC curve for cystatin C was 74.3% and the best threshold was 1.415 mg/L (sensitivity 66.7%, specificity 73.2%).* Conclusions*. Baseline cystatin C values were not able to predict renal failure during subsequent treatment. In conclusion, serum cystatin C is not a reliable early marker to efficiently predict renal failure in patients receiving chemotherapy.

## 1. Introduction

During the treatment with chemotherapeutic agents such as cisplatin or carboplatin renal function should be closely monitored, to predict the potential renal damage [1]. Nephrotoxicity of these cytotoxic agents is dose-related and cumulative; therefore, early recognition of renal damage is needed, in order to use them in a safe and effective way [2].

The glomerular filtration rate (GFR) provides the best overall estimate of renal function. It is calculated with the clearance of an exogenous substance such as inulin, iothalamate, Cr-EDTA. However, because of the cost and inconvenience, serum creatinine and creatinine clearance (CrCl) are the most widely used measures of renal function. CrCl, however, tends to overestimate GFR because creatinine is filtered by the glomeruli and secreted by the tubules, and furthermore it implies the need for a 24-hour urine collection that is obviously inconvenient for the patient and susceptible of errors [3, 4]. To make the serum creatinine-based GFR measurements more simple, some formulae such as Cockcroft-Gault have been introduced [5]. Some have reported the utility of GFR equation based on creatinine for use in oncology patients during chemotherapy treatment [6, 7].

Cystatin C, a cysteine protein kinase inhibitor, is constituted of 120 amino acids and belongs to the cystatin superfamily. It is filtrated by the glomeruli and almost completely reabsorbed and catabolized in the proximal renal tubular cells. This protein is not significantly influenced by inflammation and, differently from creatinine, it is not affected by muscle mass, sex, race, and height. In addition, creatinine assay suffers from analytical influences [8].

Several studies have shown that cystatin C is a more sensitive marker of decreased GFR than serum creatinine [9–11], but in several malignancies, the serum cystatin C levels are increased probably related to its nature as a cysteine protease inhibitor [12].

In this study we tested the predictive accuracy of the baseline serum cystatin C levels as a tool to predict the occurrence of renal failure, defined as two different levels of GFR impairment, in patients receiving platinum-based chemotherapy. We also tested whether baseline cystatin C levels could predict time to renal failure in these patients.

## 2. Materials and Methods

### 2.1. Patients

Patients affected by either lung, urologic, or gynecologic tumors, for whom a treatment with platinum-based chemotherapy, containing cisplatin or carboplatin, was indicated as eligible, were included in this study. The only specific exclusion criteria was a baseline creatinine value over the reference interval. Therefore, before treatment start, all the patients were screened for renal impairment by creatinine measurement, and patients with pretreatment renal impairment were excluded from the study.

### 2.2. Study

In all patients we measured serum cystatin C, creatinine concentrations, and calculated glomerular filtration rate (GFR) according to Cockcroft-Gault equation (the most commonly used when our study began) at baseline, before each cycle, and at the end of the last cycle according to clinical practice. For the present analysis, only baseline values of cystatin C were used.

### 2.3. Analytical Methods

Serum samples were taken in Vacutainer tube SST II Advance (BD, ref. 366468) and analyzed in less than one hour.

The serum cystatin C concentration was determined by Fluorimetric Enzyme Immuno Assay (FEIA) on AIA 360, Tosoh Bioscience, Tokyo, Japan. The cystatin C reference range was 0.52–0.97 mg/L.

The serum creatinine concentration was determined enzymatically by a spectrophotometric method on a Cobas C6000 automated analyzer, Roche Diagnostics. The creatinine reference ranges were 0.51–0.95 mg/dL for females and 0.67–1.17 for males.

GFR was calculated by Cockcroft-Gault equation GFR  [mL/min/1.73 m^2^] = 175 × [serum  creatinine  (mg/dL)]^−1.154^ × (Age)^−0.203^ × *R*.

The Cockcroft-Gault equation was used to guide carboplatin dosing.

### 2.4. Statistical Analysis

Statistical analysis was performed using S-PLUS software (S-PLUS 6.0 Professional, release 1; Insightful Corporation, Seattle, WA, USA) and SPSS software (SPSS Statistics, version 2; IBM).

In detail, to assess the predictive role of baseline cystatin C for subsequent renal failure, we used the cystatin C concentration measured before the first cycle of chemotherapy and we exploratively considered two different definitions of renal failure based on the values of GFR: below 60 mL/min/1.73 m^2^ or 80 mL/min/1.73 m^2^ during the treatment.

To assess the predictive accuracy of serum cystatin C concentration in predicting renal failure (reduction of GFR) we performed ROC curves. For each ROC curve (GFR < 60 mL/min/1.73 m^2^ and GFR < 80 mL/min/1.73 m^2^) the area under the curve (AUC) and the best threshold were identified, describing sensitivity and specificity. In detail, best threshold was calculated as the value with the highest Youden's index* J* (sensitivity + specificity − 1).

As a reference, the same analyses were performed using baseline creatinine levels.

For both definitions of renal failure (GFR < 60 mL/min/1.73 m^2^ and GFR < 80 mL/min/1.73 m^2^), patients were divided into 2 groups according to the baseline value of cystatin C (higher or lower than the best threshold) and Kaplan Meier curves were employed to evaluate the time to renal failure in the two groups.

## 3. Results

### 3.1. Patients Clinic Features

A total of 52 patients (17 men and 35 women) were included in the study. The median age was 62 years (range 41–78). Of these patients, 36 had urogynecologic cancers and 16 had lung cancer. According to the standard chemotherapy regimens used in clinical practice for the different tumors, 27 patients were treated with cisplatin and 25 with carboplatin (Table 1).

Prior to first chemotherapy administration, the median serum creatinine level was 0.72 (range 0.51–1.14), the median serum cystatin C level was 1.30 (range 0.75–1.94), and the median of CrCl calculated by the Cockcroft-Gault equation was 94.3 (range 47.0–143).

At the end of treatment the median serum creatinine level was 0.73 (0.46–1.72), the median serum cystatin C level was 1.27 (0.61–2.14) and the median creatinine clearance calculated by the Cockcroft-Gault equation was 93.2 (29.4–146) (Table 2).

### 3.2. Roc Curves

**Figure 1:**
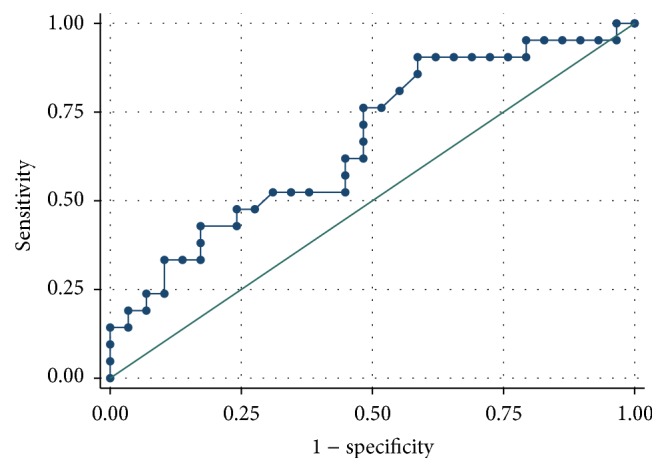
Cystatin C ROC curve for the prediction of renal failure (defined as GFR < 80 mL/min/1.73 m^2^).

**Figure 2 fig2:**
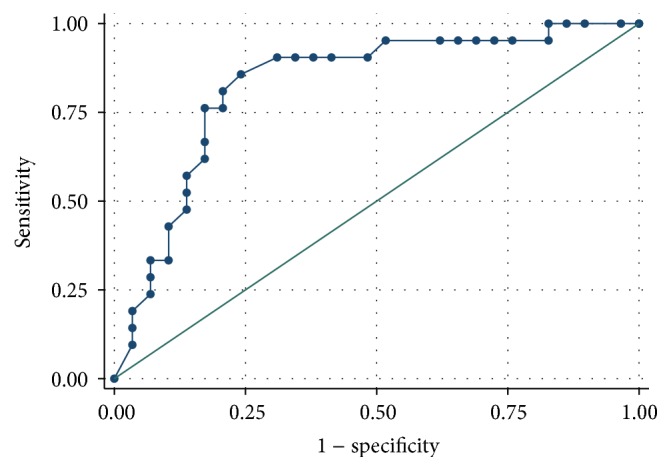
Creatinine ROC curve for the prediction of renal failure (defined as GFR < 80 mL/min/1.73 m^2^).

**Figure 3 fig3:**
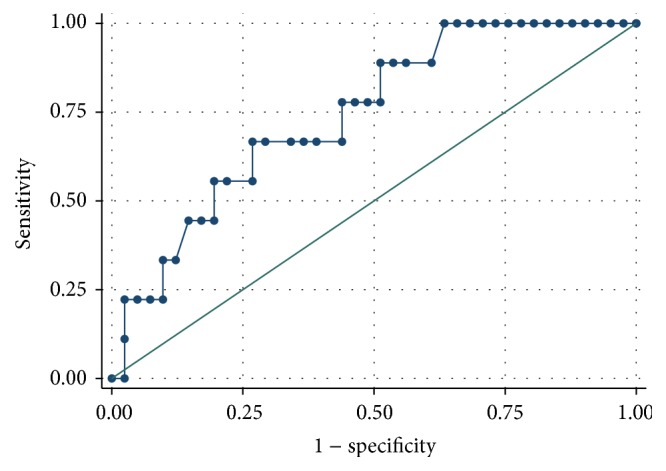
Cystatin C ROC curve for the prediction of renal failure (defined as GFR < 60 mL/min/1.73 m^2^).

**Figure 4:**
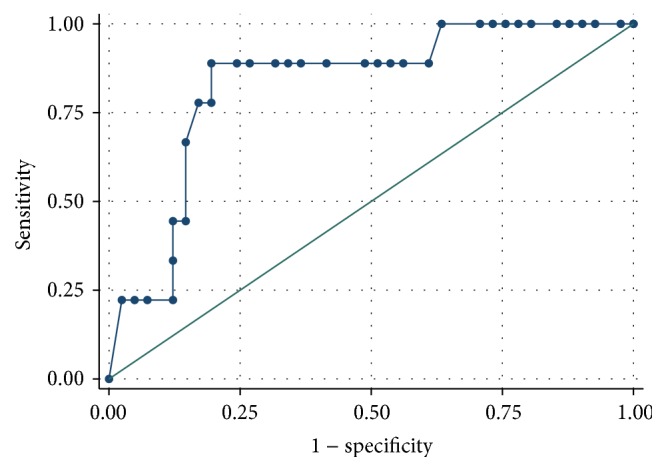
Creatinine ROC curve for the diagnosis of renal failure (defined as GFR < 60 mL/min/1.73 m^2^).

The ROC analysis demonstrated a better predictive performance for creatinine compared to cystatin C (Figures 1 [Fig fig2] [Fig fig3]–4).

When considering GFR < 80 mL/min/1.73 m^2^ during the treatment for the definition of renal failure, AUC for cystatin C was 0.667, while the AUC for creatinine was higher (0.792). The best threshold value for cystatin C was 1.135 (associated with 90.5% sensitivity and 41.1% specificity, Youden index 0.319).

When considering GFR < 60 mL/min/1.73 m^2^ during the treatment for the definition of renal failure, AUC for cystatin C was 0.743, while the AUC for creatinine was 0.818. The best threshold value for cystatin C was 1.415 (associated with 66.7% sensitivity and 73.2% specificity, Youden index 0.398).

### 3.3. Description of Time to Renal Failure

Description of time to renal failure was performed using both definitions of renal failure (GFR < 80 mL/min/1.73 m^2^ and GFR < 60 mL/min/1.73 m^2^).

**Figure 5:**
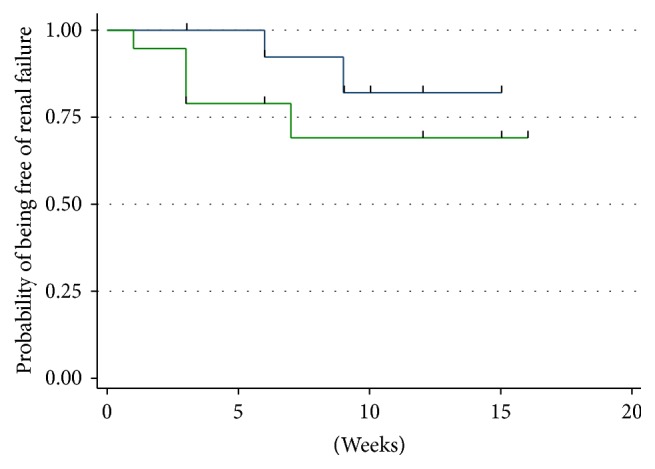
Kaplan Meier curve for the evaluation of the time to renal failure (defined as GFR < 80 mL/min/1.73 m^2^). Only patients with baseline GFR ≥ 80 mL/min/1.73 m^2^ were included in the analysis. Blue curve: patients with baseline Cystatin C < 1.135. Green curve: patients with baseline Cystatin C ≥ 1.135.

When renal failure was defined as GFR < 80 mL/min/1.73 m^2^, the best threshold of baseline cystatin C value according to Youden index (1.135) allowed dividing patients with baseline GFR > 80 mL into two groups. Seventeen patients had GFR < 80 mL at baseline and were excluded; therefore the number of patients included in this analysis was 35. Among them, a fall of GFR < 80 mL was observed in 8 cases. Time to GFR < 80 mL was not significantly different between the two groups (Figure 5).

**Figure 6:**
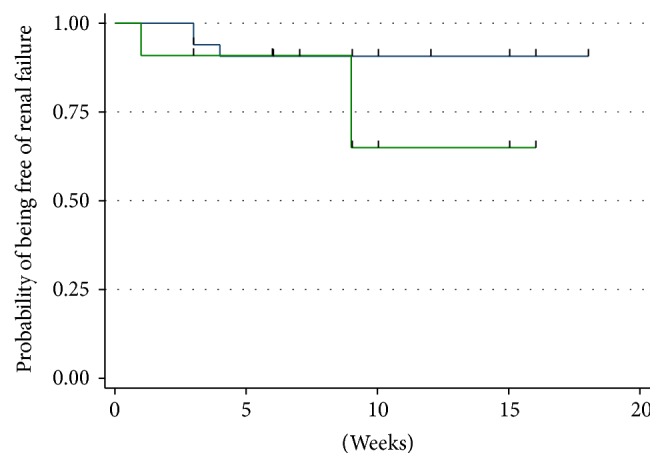
Kaplan Meier curve for the evaluation of the time to renal failure (defined as GFR < 60 mL/min/1.73 m^2^). Only patients with baseline GFR ≥ 60 mL/min/1.73 m^2^ were included in the analysis. Blue curve: patients with baseline Cystatin C < 1.415. Green curve: patients with baseline Cystatin C ≥ 1.415.

When renal failure was defined as GFR < 60 mL/min/1.73 m^2^, the best threshold of baseline cystatin C value according to Youden index (1.415) allowed to dividing patients with baseline GFR > 60 into two groups. Six patients had GFR < 60 mL at baseline and were excluded; therefore the number of patients included in this analysis was 46. Among them, a fall of GFR < 60 mL was observed during the treatment in only 6 cases. Time to GFR < 60 was not significantly different between the two groups (Figure 6).

## 4. Discussion

For the calculation of adequate doses of nephrotoxic agents like cisplatin and carboplatin, estimation of kidney function in each patient is required. The evaluation of the glomerular filtration rate (GFR) by exogenous markers is a gold standard; however it is complex, expensive, and difficult in routine clinical practice. Therefore, GFR calculation methods are mostly based on the creatinine serum level. The Cockcroft-Gault creatinine-based equation is widely applied.

Recent studies suggest that serum cystatin C may serve as a reliable alternative clinical marker of renal function [13, 14].

Our results indicate that, in cancer patients receiving platinum-based chemotherapy, baseline cystatin C serum level does not predict renal failure.

There are some limitations to our analysis. First, we used a small study population, including lung and urogynecological cancers (with the exclusion of renal cancer). On one side, this might limit generalization of our findings although we believe that the drug combinations used in this study cover a large amount of the use of platinum derivatives in clinical practice. On the other side, heterogeneity of the underlying type of cancer might be a confounding factor, although it should improve generalizability of our findings, partially contrasting limitations deriving from the small sample size. Second, we did not adjust our analysis for comorbidity and concomitant medications. However, it should be considered that our patients were all considered eligible for an active anticancer treatment based on a platinum-containing combination, limiting the scenario of possible comorbidity or concomitant treatments to the ones that do not prevent the prescription of chemotherapy in clinical practice. Third, we did not use the CKD-EPI equation for calculation of GFR values, now considered as a gold standard method. Actually, our choice was due to the fact that, at the time this study was conducted, the Cockroft-Gault formula was routinely used in our practice (and in many clinical trials). While we are aware that the performance of the Cockroft Gault equation differs from that of CKD-EPI for GFR values higher than 90 mL/min, we underline that the choice of the formula should not represent a bias of this analysis because the same formula was applied for baseline and subsequent assessments of GFR. Fourth, our analysis does not account for dynamic change of cystatin C levels. Our choice was due to the consideration that an analysis of the change of cystatin over the treatment might be confused with the outcome. In principle, if cystatin was a good biomarker, its change during treatment would be parallel to the reduction of renal function and the analysis would be biased in favour of finding a statistically significant association. For these reasons, we focused on predictive ability of the baseline values only that could be useful in clinical decision making. Finally, our analysis, due to the small number of patients and to the very limited number of renal failures during the chemotherapy, showed a fair statistical power to detect the predictive ability of baseline cystatin C values. As detailed in the results, only 6 events were recorded when considering the threshold of 60 mL/min/1.73 m^2^ for the definition of renal failure and only 8 events when considering the higher threshold of 80 mL/min/1.73 m^2^. In other words, our results, showing no difference in the time to renal failure between groups of patients divided according to baseline value of cystatin C, do not support the predictive role of cystatin C but cannot definitely rule out the potential usefulness of this test. Given that, even with cisplatin or other drugs usually considered at relevant risk of renal toxicity, the absolute number of renal impairment occurring during the treatment is reasonably limited, only studies with a very large number of patients would have enough power to demonstrate the predictive role of this (or other) biomarkers.

Furthermore, in several malignancies, the serum cystatin C levels are elevated, probably related to its nature as a cysteine protease inhibitor [15] and this phenomenon is associated with more aggressive forms of tumors [16]. Our data are consistent with those from other studies reporting the serum cystatin C levels increase in cancer patients. In fact patients that received platinum based chemotherapy do not seem to result from impaired kidney function [13, 17].

## Figures and Tables

**Table 1:** Patients characteristics.

Characteristic	Nr. of patients (%)
*Total*	52 (100)
Male	17 (32.7)
Female	35 (67.3)
*Median age*, range	62, 41–78
*Tumor type*	
Lung cancer	16 (30.8)
Urogynecological cancer	36 (69.2)
*Chemotherapy*	
*Cisplatin*	27 (51.9)
Cisplatin + gemcitabine	17 (32.7)
Cisplatin + vinorelbine	7 (13.5)
Cisplatin + Alimta	3 (5.8)
*Carboplatin*	25 (48.1)
Carboplatin + gemcitabine	4 (7.7)
Carboplatin + taxol	20 (38.5)
*Impaired renal function at the beginning of the therapy*	
GFR < 80	17 (32.7)
GFR < 60	6 (11.5)

**Table 2:** Renal function markers values.

Parameter	Measured value	
	Median	Range
*Serum creatinine, mg/dL (n)*		
Before chemotherapy (53)	0.72	0.51–1.14
At the end of the cycle (53)	0.73	0.46–1.72
*Serum cystatin C, mg/L*		
Before chemotherapy (51)	1.30	0.75–1.94
At the end of the cycle (46)	1.27	0.61–2.14
*GFR, mL/min/1,73 m* ^*2*^		
Before chemotherapy (53)	94.3	47.0–143
At the end of the cycle (53)	93.2	29.4–146
